# The impact of adhering to a quality indicator for sedation, analgesia, and delirium management on costs, revenues, and clinical outcomes in intensive care in Germany: A retrospective observational study

**DOI:** 10.1371/journal.pone.0308948

**Published:** 2024-08-15

**Authors:** Alexander Zuber, Kerstin Rubarth, Frank Förster, Felix Balzer, Claudia Spies, Daniel Fürstenau, Oliver Kumpf

**Affiliations:** 1 Commercial Department, Klinikum Rechts der Isar, Technische Universität München, Munich, Germany; 2 Institute of Medical Informatics, Charité - Universitätsmedizin Berlin, Corporate Member of Freie Universität Berlin and Humboldt-Universität zu Berlin, Berlin, Germany; 3 Institute of Biometry and Clinical Epidemiology, Charité - Universitätsmedizin Berlin, Corporate Member of Freie Universität Berlin and Humboldt-Universität zu Berlin, Berlin, Germany; 4 Charité - Universitätsmedizin Berlin, Corporate Member of Freie Universität Berlin and Humboldt-Universität zu Berlin, Corporate Controlling, Berlin, Germany; 5 Department of Anesthesiology and Intensive Care Medicine, Charité - Universitätsmedizin Berlin, Corporate Member of Freie Universität Berlin and Humboldt-Universität zu Berlin, Berlin, Germany; 6 Department of Information Systems, Freie Universität Berlin, School of Business & Economics, Berlin, Germany; Federal University of Sao Carlos: Universidade Federal de Sao Carlos, BRAZIL

## Abstract

**Background:**

Management of sedation, analgesia, and delirium influences morbidity, mortality, and quality of life in patients treated in intensive care. Assessing quality indicators as part of a quality management and assurance program is an established method to ensure process quality. Currently, there is limited research on the effect of evaluating quality indicators on economic outcomes. The aim of the study was to investigate the adherence to an indicator on management of sedation, analgesia and delirium, and explore potential effects on hospital economics and clinical outcomes.

**Methods:**

In this retrospective cohort study, we analyzed routine data from 20,220 patient records from the hospital information system of a tertiary university hospital, collected from January 2012 to December 2019. We compared two predefined subgroups with either high indicator adherence or low indicator adherence regarding factors like disease severity scores, comorbidities, and outcome measures. We used logistic regression models to examine the influence of quality indicator adherence on economic measures such as Diagnosis-related group (DRG) incomes, revenue margins, and costs, and clinical outcomes. Additionally, we used propensity score matching to probe our findings.

**Results:**

Overall revenue margins in this cohort were negative (-320€). High adherence to the quality indicator was associated with a positive revenue margin (+197€) compared to low adherence (-482€). Higher adherence was also associated with lower costs. Additionally, high adherence was associated with reduced mortality (OR 0.84, 95% CI 0.75–0.95) and reduced duration of mechanical ventilation and hospital stay (17 hours and 1 day respectively).

**Conclusion:**

Higher adherence to a quality indicator for sedation, analgesia, and delirium management was associated with economic returns and costs. We also found an association with lower mortality and reduced length of stay. Further research on these associations may help identify opportunities for quality improvement without increased resource use.

## Introduction

Quality management in the intensive care unit (ICU) involves evaluating the structure, process, and outcomes of care. Process performance is especially important because patient factors can significantly influence outcome measures [1]. To assess the quality of care, it is necessary to regularly measure and evaluate quality indicators (QIs). This is considered a crucial step in the continuous improvement of care [2]. In many countries QIs are used for intensive care [3]. In Germany, the German Association for Intensive Care and Emergency Medicine (DIVI) publishes QIs for the ICU on a regular basis, approximately every 4 years [4]. They consist of 10 indicators, predominantly assessing processes of care. Among these QIs one is focused on management sedation, analgesia, and delirium scores. The purpose of this indicator is to screen for sedation depth, level of pain and presence of delirium. Every single item uses a screening indicator that is measured routinely every shift separately or as part of a treatment bundle [4].

Managing sedation, analgesia, and delirium is a vital process in the ICU. Consistent and systematic screening is essential to identify and treat these symptoms properly. The use of validated instruments to measure and detect sedation, analgesia, and delirium is recommended in international guidelines and is an important part of providing evidence-based care [5,6]. Guideline-based management needs consistently documented monitoring and is considered a key prerequisite for adequate therapy. However, compliance with these recommendations is often not sufficient.

Inadequate management of sedation, analgesia and delirium represents a risk factor for mortality in the ICU [7]. It also negatively influences physical and cognitive function following intensive care treatment [8]. A recent meta-analysis found that delirium was associated with prolonged hospital stay and increased costs. One reason for this was the poor recognition of this state [9]. Adherence to treatment standards for sedation, analgesia, and delirium improves patient-related outcomes and reduces costs [10]. However, there are open questions regarding socio-economic effects of better treatment quality on a hospital level (i.e., cost-effectiveness).

Previous research has shown an impact of QI adherence on economic outcomes, specifically in management of sedation, analgesia, and delirium, and also for weaning from mechanical ventilation [11,12]. These studies served as the basis for our models and discussions in this study. Our hypothesis was that better performance in the management of sedation, analgesia, and delirium as measured by high QI adherence, could be associated with improved clinical outcomes and positive effects on revenue margins.

## Materials and methods

### Data sources

For this single centre, retrospective observational study, data were collected from the electronic records of patients treated in the five ICUs, associated with the department of anaesthesiology and operative intensive care at Charité –Universitätsmedizin Berlin, a tertiary university hospital. The study included patients who were admitted and discharged between January 1, 2012, and December 31, 2019. All patients included in the study were over 18 years of age and had a length of stay in the ICU of more than 24 hours. Only patients with a single ICU stay per hospital stay were included in the study because multiple stays are combined according to coding rules in Germany potentially leading to bias of economic results. Therefore, we report no ICU readmission rate as a measure of complications during the ICU stay.

All patients in the ICU received routine treatment according to clinical practice guidelines and the department’s internal standard operating procedures.

Data were collected from electronic health care records and included solely data on routine patient care and standard data for administrative purposes.

Data on sedation, analgesia, and delirium management were derived from the ICUs patient data management system (PDMS; Computer Organised Patient Report Assistant = COPRA System, Berlin, Germany). These data were entered into the PDMS automatically from patient monitors and manually by caregivers. The ICU staff were responsible for controlling and validating PDMS data as part of the routine documentation process. The design of the PDMS prevents manual alterations to the data after discharge.

Adherence to quality indicators was measured by exporting the data to a separate database, which was not part of the clinical routine. These data were primarily used by the department’s quality management team. PDMS data were in part transferred to the hospital information system (HIS) (SAP IS-H, SAP Walldorf, Germany) for case revenue calculation that also allowed case cost analysis. Basic demographic data, clinical and administrative parameters of in-patient cases (e.g., length of stay) were available from the HIS.

All data were retrieved using a structured query. Following this step all case specific data were aggregated, exported and pseudonymized immediately thereafter for this study between July 1^st^ 2022 and December 1^st^ 2022 and stored on a local server with access only to the investigators of the study. Only data necessary for this study were extracted.

We excluded incomplete patient datasets (PDMS and or HIS) and cases that had a cost-revenue difference exceeding 50,000€ where we expected abnormal case coding or additional payments considering German remuneration rules.

The ethics committee of the Charité approved this data analysis (EA2/079/22) prior to the analysis. Due to the retrospective design of the study using data from standard care the need for informed consent was waived. The data protection officer of the Charité advised on data protection rules prior to the analysis to ensure compliance with those rules. Data were anonymized and only the principal investigators had access to original patient data on protected internal servers. This original research is in accordance with the Consolidated Health Economic Evaluation Research Standards. The study was registered at Trial registration: NCT05384613.

### Measures

Demographic data included age, sex, type of ICU admission, and disease severity scores upon admission. We used the Charlson Comorbidity Index (CCI) calculated based on ICD-10 codes, as described in [13].

Adherence to the quality indicator was assessed based on the most recent version of the German Quality Indicators, with each item in the quality indicator on sedation, analgesia, and delirium being measured and documented in the PDMS at least once per 8-hour shift. According to adherence to this QI, we divided the cohort into two groups based on the number of measurements performed: the high-adherence group (HAG) included patients with 8 or 9 completed measurements out of a possible 9 for a valid score for sedation/agitation, pain, and delirium screening each shift, while the low-adherence group (LAG) included patients with 7 or fewer measurements. Mean adherence was defined as number of completed measures divided by the number of possible measures, resulting in a ratio (number between 0 and 1.0). High adherence was defined by a ratio ≥8/9^th^, and low adherence by a ratio <8/9^th^. 1 missing value is considered acceptable also leading into the high adherence group 8 x 1/9^th^ ≥8/9^th^. All remaining patients were considered in the low adherence group (<8/9^th^). In both groups, the number of measured score values for the complete treatment period was divided by the number of potential score values. An overview over the scoring method and the scores used are presented in S1 Fig.

Revenues were calculated according to the German reimbursement rules based on DRG catalogues and defined coding rules (G-DRG) (https://www.g-drg.de/das-institut) for the year in which each patient was treated, determined by the admission date. Any additional revenues from intra- or extrabudgetary sources were included, such as revenues for expensive treatment modes or drugs or blood products, all in accordance with these rules. Costs were calculated using data from the hospital’s HIS. Margins were calculated by subtracting the calculated costs from the reimbursement rates. An overview is provided in S2 Fig.

### Statistical analysis

We analyzed the data using descriptive statistics, employing corresponding statistical measures (mean and standard deviation for metric demographic variables, median and interquartile range for metric and ordinal outcome variables and absolute and relative frequencies for categorical data). To evaluate the impact of belonging to the HAG on economic and clinical outcomes and account for potential confounding variables, we conducted multiple linear as well as logistic regression models, depending on the scale of the outcome. Potential confounding variables were selected a priori based on prior expert knowledge of the associations with the respective outcomes. Considering potential confounders, we accounted for age, sex, and CCI (age-adjusted) of patients, along with ICU admission type (emergency surgery, medical emergency and elective surgery), the year of hospital stay, and the group of diagnoses to which the main diagnosis was related. The diagnoses groups included cardiac, malignant, neurological, pulmonary, sepsis, trauma, cerebral diagnoses, and a group termed ‘other diagnoses’, encompassing all remaining diagnosis groups. The assignment of the individual main diagnosis to the diagnosis group was performed based on the first three digits of the International Classification of Diagnoses Version 10 (ICD-10) codes of the main diagnosis. We used R^2^-values to assess the goodness of fit for the respective models. However, due to the complexity and heterogeneity of patients in the ICU, we did not anticipate obtaining high R^2^-values. As a sensitivity analysis we employed propensity score analysis using the nearest neighbour matching algorithm. The matching variables were identical to the potential confounding variables included in the regression models. The quality of the matching was evaluated using standardized mean differences and is reported in detail in the supporting information. We then used two-sample Wilcoxon tests to test for group differences between the matched cohorts. Statistical analysis was conducted using SPSS Version 21 (IBM Corp.) and R version 4.2.1 (R Core Team 2022). A two-tailed p-value of <0.05 was considered statistically significant. Due to the exploratory design of this study, no adjustment of p-values and confidence intervals was conducted. Hence, p-values and confidence intervals need to be interpreted as hypothesis-generating.

## Results

### Patient demographics

**Fig 1:**
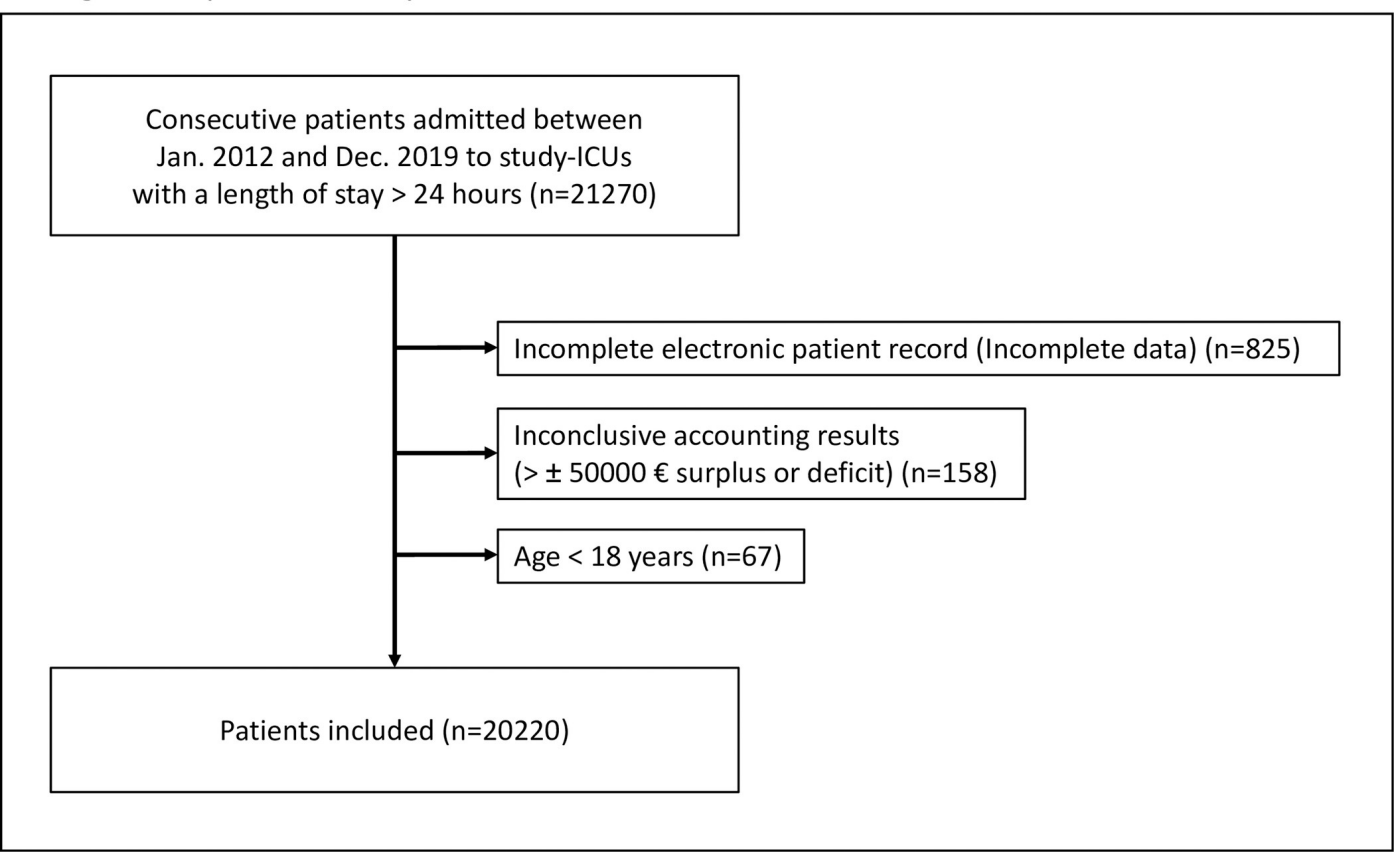
CONSORT diagram of the study.

We screened 21270 patient records for eligibility and excluded 1050 patients, which led to a final cohort of 20220 patients. A CONSORT diagram of the study is shown in Fig 1. Patient demographics are presented in Table 1. During the study, between 1992 (9.9%) and 2882 (14.3%) patients were treated in the department annually.

**Table 1:** Patient demographics of the study cohort and differences between high adherence (HAG) and low adherence group (LAG).

	All patients	QI adherence groups*	
		**HAG**	**LAG**
	n = 20220	n = 4879	n = 15341
**Demographics **			
Age in years ^a^	64.6 (36.9)	64.1 (22.8)	64.8 (22.0)
Male sex (%)	11780 (58.3)	2954 (60.5)	8826 (57.5)
*Admission type (%)*			
Elective surgery	7159 (35.4)	2437 (49.9)	4722 (30.8)
Emergency surgery	4349 (21.5)	908 (18.6)	3441 (22.4)
Medical	8712 (43.1)	1534 (31.4)	7178 (46.8)
*Score on admission*			
APACHE II	15 [10;22]	14 [9;22]	15 [10;21]
SAPS II	34 [24;49]	34 [24;50]	35 [24;48]
CCI (age-adjusted)	5 [3;8]	5 [3;7]	5 [3;8]
**Adherence to quality indicator**			
QI overall	0.79 [0.63;0.89]	0.94 [0.92;0.99]	0.73 [0.56;0.81]
QI sedation	0.81 [0.63;0.92]	0.97 [0.90;1.0]	0.75 [0.50;0.85]
QI pain	0.81 [0.67;0.91]	0.97 [0.91;1.0]	0.75 [0.58;0.83]
QI delirium	0.78 [0.57;0.90]	1.00 [0.91;1.0]	0.70 [0.5;0.82]
**Outcome parameter**			
Patients with MV (%)**	14637 (72,4%)	3698 (75,8%)	10939 (71,3%)
Duration of MV (hours)	45 [12;163]	34 [11;95]	51 [12;196]
LOS study ICU	3.7 [1.9;8.0]	3.6 [1.8;6.6]	3.8 [1.9;9.0]
LOS hospital	14 [8;23]	13 [8;20]	14 [8;24]
Death ICU	2024 (10%)	351 (7.2%)	1673 (10.9%)
Death hospital	2590 (12.8%)	448 (9.2%)	2142 (14.0%)
Readmission hospital	725 (3.6%)	543 (3.5%)	182 (3.7%)
**Economic outcomes**			
DRG income (in €)	17080 [8503;32397]	17348 [10156;27942]	17010 [8156;33133]
Total costs (in €)	17815 [10477;30657]	16820 [11349;25624]	18415 [10208;32536]
Revenue margin (in €)	-320 [-3928;4840]	197 [-3236;5277]	-482 [-4193;4679]

*threshold value ≥ 8/9

**patients with at least 1 hour of mechanical ventilation (MV); percent of complete cohort or respective adherence group; All values presented as median with interquartile range, except where indicated. ^a^Mean value with standard deviation; SAPS = Simplified Acute Physiologic Score; CCI = Charlson Comorbidity Index. DRG = Diagnosis related groups.

### Quality indicator adherence

In the next step, we analyzed adherence to the examined QI. The calculation for QI adherence is shown in S1 Fig, while the results for QI adherence in the studied cohort are presented in Table 1. After dividing the cohort in high adherence group (HAG) and low adherence group (LAG), we observed differences between the two groups. In the LAG, the median value for overall adherence was 0.73 (0.56–0.81; 95%-CI) compared to 0.94 (0.92;0.99) in the HAG. Regarding the 3 subcategories of QI adherence, the LAG achieved 0.70 (0.5;0.82) for delirium screening, 0.75 (0.58;0.83) for pain monitoring, and 0.75 (0.50;0.85) for sedation monitoring, compared to the HAG, where the values were 1.00 (0.9;1) for delirium, 0.97 (0.91;1) for pain, and 0.97 (0.9;1) for sedation, respectively. The values for the LAG were notably below the threshold value of 8/9^th^.

### Economic and clinical results in the study population

In the first step, we analyzed our main economic and clinical parameters in the complete study cohort. These data are also presented in Table 1. Overall total costs per patient were 17815€ [10477;30657] and DRG income was 17080€ [8503;32397] per patient. This resulted in a negative revenue margin of -320€.

Clinical observations showed an overall length of stay (LOS) in the hospital of 14 [8;23] days, with a LOS in the intensive care unit (ICU) of 3.7 [1.9;8.0] days. Mechanical ventilation was required for 72.4% of all patients, with a median duration of 45 [12;163] hours in those patients. Regarding potential complications, we noted a hospital readmission rate of 3.6%.

We then examined the presence of differences in the main demographic data between the HAG and the LAG. We observed several differences in the two groups, such as sex distribution, type of admission and the rate of mechanically ventilated patients differed, with 75.8% in the HAG versus 71.3% in the LAG (Table 1).

Overall, we observed a considerable number of patients who were not monitored as recommended by the German ICU quality indicator regarding management of sedation, analgesia, and delirium. Additionally, a difference in admission type was noted, with more patients from elective surgery in the HAG. This first exploratory analysis showed differences in economic and outcome results which will be presented in the following paragraphs. Detailed results of the differences between the groups are also presented in Table 1.

In a next step, we employed a logistic regression model to analyze which of these influence factors were potentially associated with higher or lower QI adherence. In the initial descriptive results, we saw higher adherence (49.9%) in elective admission patients. This was confirmed in the regression model which showed a markedly lower odds for medical patients (OR 0.61, 95% CI: 0.57 to 0.66) or patients following emergency surgery (OR 0.92, 95% CI: 0.84–1.00) to be in the HAG. Further results of this analysis are shown in S1 Table.

### Influence of high and low adherence group on economic outcomes

In the HAG, a net loss (i.e., negative revenue margin) was observed in 2,653 patients (48.9%) compared to 7,955 (53.8%) in the LAG group (p<0.001). Overall, in the LAG, we found a larger difference between cost and DRG income, resulting in an overall negative revenue margin (-482€ [LAG] versus +197€ [HAG]). This is also shown in Table 1. However, this effect was not confirmed in the logistic regression analysis (Table 2, left column). Here we couldn’t confirm an association with high QI adherence. Nonetheless, other factors were apparent for positive or even revenue margins, such as elective surgical admission and a cardiac main diagnosis. We also analyzed which factors would influence achieving a net zero or a positive revenue margin. An overview over the results is provided in Table 2. We further analyzed the extent of the revenue difference between the groups. Although there were lower chances for almost all groups compared to the reference groups, definite values differed substantially, showing, for example, higher DRG income values for emergency surgery despite the overall odds being low to achieve this (Table 2). We found an increase in positive or even revenue margins over accounting years. This analysis was primarily conducted to control for changes in reimbursement schemes over the years. While such changes are a possible explanation of changes in positive or even margins, a potential interaction with other factors is also thinkable.

**Table 2:** Regression models for economic outcome.

	*Logistic regression*		*Linear regression*	
	Positive or even margin		DRG income	
*Predictors*	*Odds Ratios*	*CI*	*Estimate (€)*	*CI*
(Intercept)	0.89	0.74–1.06	26051.18	23464.41–28637.95
High adherence group	1.03	0.96–1.11	-4694.43	-5708.55 –-3680.32
Age	1.00	1.00–1.01	-353.77	-384.40 –-323.15
Male gender	1.07	1.01–1.14	2850.33	1983.93–3716.72
SAPS-2 on admission	1.01	1.00–1.01	319.99	293.07–346.90
CCI (Age adjusted)	1.01	1.00–1.02	2397.04	2284.65–2509.42
*Admission type* *				
Emergency Surgery	0.92	0.84–1.00	6264.17	4996.77–7531.56
Medical	0.61	0.57–0.66	-2198.87	-3252.91 –-1144.83
*Main diagnostic category* **				
Infection, sepsis	0.38	0.33–0.45	-1960.24	-4237.14–316.66
Malignant	0.40	0.36–0.44	-12748.21	-14209.41 –-11287.01
Pulmonary	0.74	0.65–0.84	6735.38	4751.06–8719.71
Other	0.32	0.29–0.35	-4312.81	-5588.43 –-3037.18
Trauma	0.30	0.26–0.34	-8741.70	-10738.85 –-6744.56
Cerebral	0.56	0.51–0.61	-9873.00	-11222.49 –-8523.50
Year				
2013	0.88	0.78–0.99	516.77	-1197.92 – 2231.45
2014	1.33	1.17–1.51	1908.18	49.11 – 3767.25
2015	1.60	1.43–1.81	3940.43	2218.85 – 5662.02
2016	1.37	1.21–1.54	3981.73	2267.61 – 5695.86
2017	1.60	1.41–1.81	6078.51	4286.16 – 7870.87
2018	1.47	1.30–1.65	1185.47	-548.55 – 2919.49
2019	1.66	1.48–1.87	2135.15	436.38 – 3833.93
	Observations	20220	Observations	20220
	R^2^ Tjur	0.081	R^2^ / R^2^ adj.	0.162 / 0.161

*reference: Elective surgery

**reference: Cardiac.

To corroborate our findings, we performed propensity score matching for our main findings of the regression analysis. We compared 4633 cases from the two groups that had all necessary data available. Case matching factors were identical to the confounding variables in the regression models. This analysis confirmed the results of the regression analyses. There was no statistically significant difference between a positive or even revenue margin in the high and low adherence group when directly comparing the two groups. However, we found a difference in DRG income between the high adherence and low adherence group (S4 Fig). The detailed results are shown in S5 Table and S4 Fig. Matching categories and results are shown in S6 Table.

### Cost analysis

We also attributed various cost elements such as personnel costs or ICU costs in our analysis and compared low and high adherence groups. We found no difference between the two groups concerning cost distribution between personnel cost and materials or other costs in the regression models (S2 Table). However, when comparing propensity score matched groups, costs across all items were lower in the HAG. This leads to the assumption that higher adherence to QI was associated with reduced costs. This was true for both personnel cost and overall ICU costs. This more detailed cost analysis is provided in S4 Table. However, these results do not preclude other forms of increased resource use or additional personnel strain due to higher workflow density.

### Influence of high and low adherence group on clinical outcomes

The next step in our analysis compared the QI adherence groups to measures of clinical outcome. As one main clinical result of our study, we noted a decrease in mortality in patients with high adherence. In this group, 4918 out of 5424 patients (90.7%) survived, compared to 12712 of 14798 (85.9%) patients in the low adherence group. The logistic regression model confirmed the results of our descriptive statistics, showing a statistically significant correlation between high QI adherence with survival. In our analysis, patients in the HAG group were less likely to die (OR: 0.84, CI 0.75–0.95); p = 0.004). This effect was also observed in propensity score matching (S5 Table). We also observed a reduced length of stay in both the ICU and the hospital overall (LOS ICU -1.6 days in the HAG (CI: -1.93 –-1.23; p<0.001). See Tables 3 and S3. We also found an increase of adherence over time in our analysis. As is shown in Table 3, there is no correlation of this trend with the outcomes we observed.

**Table 3:** Regression models for clinical outcomes.

	*Logistic regression*		*Linear regression*	
	Mortality		LOS ICU	
*Predictors*	*Odds Ratios*	*CI*	*Estimate (days)*	*CI*
(Intercept)	0.00	0.00–0.01	5.91	5.01–6.80
High adherence group	0.84	0.75–0.95	-1.58	-1.93 –-1.23
Age	1.01	1.00–1.01	-0.10	-0.11 –-0.09
Male gender	1.13	1.03–1.24	1.11	0.81–1.41
SAPS-2 on admission	1.04	1.03–1.04	0.11	0.10–0.12
CCI (Age adjusted)	1.10	1.09–1.11	0.62	0.59–0.66
*Admission type* *				
Emergency Surgery	2.46	2.12–2.86	3.02	2.58–3.46
Medical	2.78	2.43–3.19	1.48	1.11–1.84
*Main diagnostic category* **				
Infection, sepsis	2.22	1.81–2.71	2.03	1.25–2.82
Malignant	1.30	1.10–1.54	-4.05	-4.55 –-3.54
Pulmonary	2.57	2.14–3.07	2.96	2.28–3.65
Other	1.23	1.06–1.43	-0.49	-0.93 –-0.04
Trauma	1.01	0.79–1.29	-0.58	-1.27–0.11
Cerebral	2.31	2.01–2.67	0.32	-0.15–0.78
Year				
2013	1.06	0.89 – 1.26	-0.49	-1.46 – 0.48
2014	0.99	0.81 – 1.20	-1.13	-2.19 – -0.08
2015	0.82	0.68 – 0.98	-1.43	-2.40 – -0.46
2016	0.84	0.71 – 1.01	-1.78	-2.75 – -0.81
2017	0.86	0.72 – 1.04	-0.74	-1.75 – 0.28
2018	0.87	0.73 – 1.04	-2.60	-3.58 – -1.62
2019	0.82	0.69 – 0.98	-3.28	-4.24 – -2.32
	R^2^ Tjur	0.147	R^2^	0.135
	Observations	20,220	Observations	20,220

*reference: Elective surgery

**reference: Cardiac.

## Discussion

In this large retrospective study, we demonstrated that compliance with quality goals, represented by adherence to a quality indicator, is associated with lower total costs and a higher revenue margin in the descriptive analysis. The regression analysis could not detect an influence of high indicator adherence on positive or even margin, which could mean, that reaching a positive margin needs more than cost reduction. However, a positive economic effect with high adherence remained intact after propensity score matching. Here we detected a trend towards reduced overall costs but also DRG income between the high adherence and low adherence group (S4 Fig) and there was no difference between occurrence of a positive or even revenue margin in the high and low adherence group (S5 Table).

As a second result high adherence was associated with a relevant reduction in mortality and reduced length of stay in this representative cohort of ICU patients.

Previous research has demonstrated an effect of quality management on economic outcomes. In one study, weaning from mechanical ventilation was associated with economic outcomes and showed that high adherence to this single QI was associated with better clinical outcome and improved economic returns [12]. Another study also addressed sedation, analgesia, and delirium, and showed that adherence to adequate monitoring was associated with shorter hospital stay, ICU LOS, duration of mechanical ventilation in primary analysis, but was associated with a decreased case mix per day and profit per day, which was not confirmed in a multiple linear regression analysis in that study [11]. This difference could partly be explained by the lower number of included patients and a different economic outcome selected.

Reasons for better clinical outcomes might include better management of sedation, analgesia, and delirium, which is part of bundles for prevention for delirium and cognitive dysfunction post-ICU treatment. This bundle approach has been shown to reduce costs [10]. Our study also showed lower costs in the HAG, but our cost analysis was not as detailed as the referenced study.

Furthermore, increasing adherence to quality indicators might be a reasonable approach to improve overall treatment quality. Since we could not test for other quality measures, i.e., other DIVI-quality indicators in this cohort, a possible interaction between them is possible. Further research should address this fact. Other approaches like telemedicine have shown to support quality improvement by increasing QI adherence [14,15].

In this study, we also associated the impact of adherence to a process-based quality indicator on clinical outcomes with significantly reduced mortality and shorter length of stay in the ICU and the hospital. Our initial exploratory analysis indicated a nearly 5% decrease in mortality, a finding corroborated by logistic regression models and propensity-score matched analyses. To our knowledge, our study is one of the first to show that the fulfilment of a quality goal was associated with significant improvement in clinical outcome parameters. The main difference was that a previous study with a similar focus involved a much smaller cohort of patients for analysis [11]. However, the quality goal was also more ambitious compared to the previous study, with 70% adherence versus over 88.8% in this study.

Although our analysis showed a marked association between outcome and adherence to a sedation, analgesia, and delirium quality indicator, we cannot exclude other factors that might have influenced this association. Outcomes such as mortality and LOS may be related to other aspects of care, like quality of infection therapy or ventilation strategies [16,17]. This could be a limitation of this study.

Additionally, it is unclear why certain patient groups were more likely to be in the LAG. Different ICUs may employ different standards for measuring, but since this study was conducted within the ICUs of one department with strong emphasis on uniform standards, strong differences between single ICUs were less likely, but we did not control for that. In our view it is unlikely that patient inherent factors like sepsis, malignant disease, or emergency admission influenced adherence to the quality indicator. Our statistical approach supports the assumption that patient factors were not likely to be responsible for measurement frequencies. Further studies have to address this fact. In our view, structural reasons for lower process adherence or, for example, insufficient staffing levels have to be analyzed as well as knowledge among staff members.

As a second relevant result of our analysis, we show that these clinical results co-occured with changes in economic results, as represented by a lower revenue margins in the low adherence group. This could mean, that this quality measure could contribute to positive clinical outcome without decreasing revenue by increased resource use. However, results from the propensity score matching suggest a more nuanced interpretation where some variance in costs may be explained by different characteristics in the low and high adherence groups, while some difference in total costs and DRG income remained after the matching. As shown in a previous study on this topic [11] the minimal effect on revenue shows there is still no mechanism that could support better process adherence by improving revenues. On the other hand, our cost analysis showed a trend towards decreased cost in the HAG. This might indicate no additional resource use, but this analysis was not detailed enough to draw robust conclusions about the cost of sedation, analgesia, and delirium monitoring. Additionally, we did not differentiate cost factors outside the ICU. This could have influenced our results as well.

This is also corroborated by the fact that personnel cost and overall ICU cost were not increased in the HAG as shown in S4 Table. Increased overall cost may also be related to a longer ICU or hospital stay, but we found no association of daily cost with any of these outcome measures (data not shown).

Improved clinical and economic results could also have other reasons, such as disease severity and resource use in different patient groups. However, our approach using logistic regression showed that patients in the HAG had a higher chance to survive. No other factor in our analysis contributed stronger to this result.

We were also able to show improved quality adherence over time (S3 Fig). Over the study period the number of patients in the LAG decreased. This might have reduced the chance to have a negative clinical outcome. Our regression analysis suggests that the treatment year itself may not affect economic results (except 2013; Table 2). However, with increasing adherence, we saw an increase in revenues and a higher probability of positive margin (Table 3 and S3 Fig). It is not clear if adherence to the QI contributed to these revenue effects over the years.

Treatment in intensive care units constitutes approximately 20% of costs in German hospitals, indicating that process improvements could be beneficial for hospital management as well [18]. Thus, improving the quality of care through improved management of sedation, analgesia, and delirium is of interest not only for the economic success of single hospitals, but also for the healthcare system at large. We found no international studies presenting comparable results. Our results hint towards a need for a change in incentives for hospital management to support quality improvement in sedation, analgesia, and delirium management.

It is currently not understood how process-based quality indicators and standard recorded outcome indicators like LOS interact and whether this has an economic impact on a hospital [19]. As our analysis shows, costs in the domains we observed like personnel and material were lower in the HAG compared to the LAG. If the overall cost reduction was due to less treatment days is unclear. Further analyzing the cost structure especially in the ICU may help identify revenue patterns to support treatment processes and, hence, outcome quality.

In the future automated measurement of scores through data collection of specific symptoms regarding sedation, analgesia, and delirium could also be a cost-effective strategy to improve quality [20].

Revenue generation in the German DRG system is unique, making it challenging to compare results internationally. Additionally, there is no consensus regarding cost measurements for intensive care [21]. We conducted this study in Germany, but we assume that these results could be relevant in other countries as well, since DRG-oriented revenues are widespread worldwide [22].

Overall, our economic results are generally encouraging, at least for the German system, as improved quality was associated with reduced cost and with better revenues, even with shorter ventilation times, that are important for revenue generation in ICUs but also with respect to resource use. Further analyses are needed to help understand these economic effects better.

One possibility to increase adherence to this or other indicators could be linked to improved revenues independent from the reimbursement system (pay for excellence) instead of sanctioning insufficient behaviour by cutting revenues (pay for performance). The latter may lead to unwanted consequences when the cost/sanction relation is tilted towards only minor losses for an institution which is not in compliance [23].

This analysis may also provide insight into alternative cross-treatment outcome indicators. These should be evaluated not only in terms of patient-oriented outcomes, but also in terms of economic outcomes as a positive incentive for service providers.

In future research causal factors for low adherence and the clinical effects of low adherence to screening standards should be studied.

Given the ongoing discussions on adjustments to reimbursement systems, we recommend considering the inclusion of quality indicators in the cost reimbursement calculation to ensure that intensive care medicine is provided at the highest quality levels possible across all hospitals. Especially incentivising quality measures like indicator adherence could be a chance to expose patients to positive behaviour by increasing revenues for these measures (with stable costs) and not to sanction insufficient behaviour as has been seen with pay-for-performance initiatives. A current initiative to regulate intensive care centres does not consider QIs so far. Still, it represents a step towards securing quality in ICUs.

### Strengths and limitations of this study

There are several strengths to our study. First, we used reliable data sources. Second, we analyzed the total actual costs, not just the budget costs per case. Third, we had access to an extensive database from a department that places a strong emphasis on adherence to standards and guidelines.

However, our study also has some limitations. First due to the structure of our database and the availability of additional data we were not able to control for other quality measures, i.e., other quality indicators of DIVI. Secondly, we also had no treatment data available, i.e., medications. Therefore, we cannot conclude an effect of potentially improved diagnosis on therapy. Previous internal analysis in routine quality management showed good adherence to treatment protocols (data not shown). The overall exploratory nature of our study that used routine data from a clinical information system, the quality of which depends on the level of documentation. Another limitation is that our economic analysis is only directly applicable to the German healthcare system, due to the use of the G-DRG system.

Finally, our analysis focussed on well-established ICU and hospital outcomes like mortality or LOS with regard to economic results. Future studies should emphasize measures of ‘quality of life’ following an ICU or hospital stay [24].

## Conclusions

In conclusion, our study found that adherence to a QI with a focus on sedation, analgesia, and delirium management is both important for clinical and economic outcomes. Our analysis also shows that hospitals have equal costs in the ICU when complying with such an indicator. However, there are no additional financial incentives or benefits. As one result it should be considered to improve benefits to incentivise good quality further.

## Supporting information

S1 Fig Principle of quality indicator measurement and scores used. (PDF)

S2 Fig Principles of revenue generation in German hospitals. (PDF)

S3 Fig Development of QI adherence over the studied period. (PDF)

S4 Fig Results for economic and clinical outcome following propensity score matching (n = 4633). (PDF)

S1 Table Influence factors for high adherence (multiple logistic regression). (PDF)

S2 Table Influence factors on overall cost (linear regression). (PDF)

S3 Table Influence factors on length of hospital stay (linear regression). (PDF)

S4 Table Cost items per case in € after propensity score matching (n = 4633). (PDF)

S5 Table Results for economic and clinical outcome following propensity score matching (n = 4633). (PDF)

S6 Table Overview over the propensity score matching categories and results. Algorithm used: Nearest neighbor.
(PDF)
