# Supplementary material for: The impact of adhering to a quality indicator for sedation, analgesia, and delirium management on costs, revenues, and clinical outcomes in intensive care in Germany: A retrospective observational study
Source: PLoS One. 2024 Aug 15;19(8):e0308948. doi: 10.1371/journal.pone.0308948 (PMC11326618; doi:10.1371/journal.pone.0308948)
Supplement: S1 Fig — (PDF) [file pone.0308948.s001.pdf]

S1 Figure: Monitoring scheme defining high and low adherence to the quality indicator

Duration	Treatment day (24 hours)						Comment
	Shift 1		Shift 2		Shift 3		Scores in use:
Pain score measurement	yes	no	yes	no	yes	no	NRS, BPS, VAS
Sedation score measurement	yes	no	yes	no	yes	no	RASS
Delirium score measurement	yes	no	yes	no	yes	no	CAM-ICU, Nu-DESC, ICDSC

Legend: Full adherence is defined as one measurement per score per shift (a partial ratio of 1/9th per measurement) during one treatment day. 1 missing value is considered acceptable also leading into the high adherence group 8 x 1/9th >= 8/9th (i. e. the goal is to reach 8 or 9 green fields). All remaining patients were considered in the low adherence group (< 8/9th). To define the adherence group (LAG or HAG) the number of measured score values for the complete treatment period was divided by the number of potential score values. NRS = numeric rating scale, BPS = behavioural pain scale, VAS = visual analog scale; RASS = Richmond Agitation Sedation Scale; CAM-ICU = Confusion Assessment Method – Intensive Care Unit, Nu-DESC = Nursing Delirium Detection Scale; ICDSC = Intensive Care Delirium Screening.
