# Supplementary material for: The impact of adhering to a quality indicator for sedation, analgesia, and delirium management on costs, revenues, and clinical outcomes in intensive care in Germany: A retrospective observational study
Source: PLoS One. 2024 Aug 15;19(8):e0308948. doi: 10.1371/journal.pone.0308948 (PMC11326618; doi:10.1371/journal.pone.0308948)
Supplement: S2 Fig — (PDF) [file pone.0308948.s002.pdf]

S2 Fig. Principles of revenue generation in German hospitals

$$\sum \text{relative weights} \times \text{Federal state base rate} = \text{DRG revenue}$$

$$\text{DRG revenue} + \text{Supplementary charges} \pm \text{Surcharges/Deductions} = \text{Hospital revenue}$$
