# Supplementary figures and images for: The impact of adhering to a quality indicator for sedation, analgesia, and delirium management on costs, revenues, and clinical outcomes in intensive care in Germany: A retrospective observational study
Source: PLoS One. 2024 Aug 15;19(8):e0308948. doi: 10.1371/journal.pone.0308948 (PMC11326618; doi:10.1371/journal.pone.0308948)

S3 Fig. Development of QI adherence over the studied period

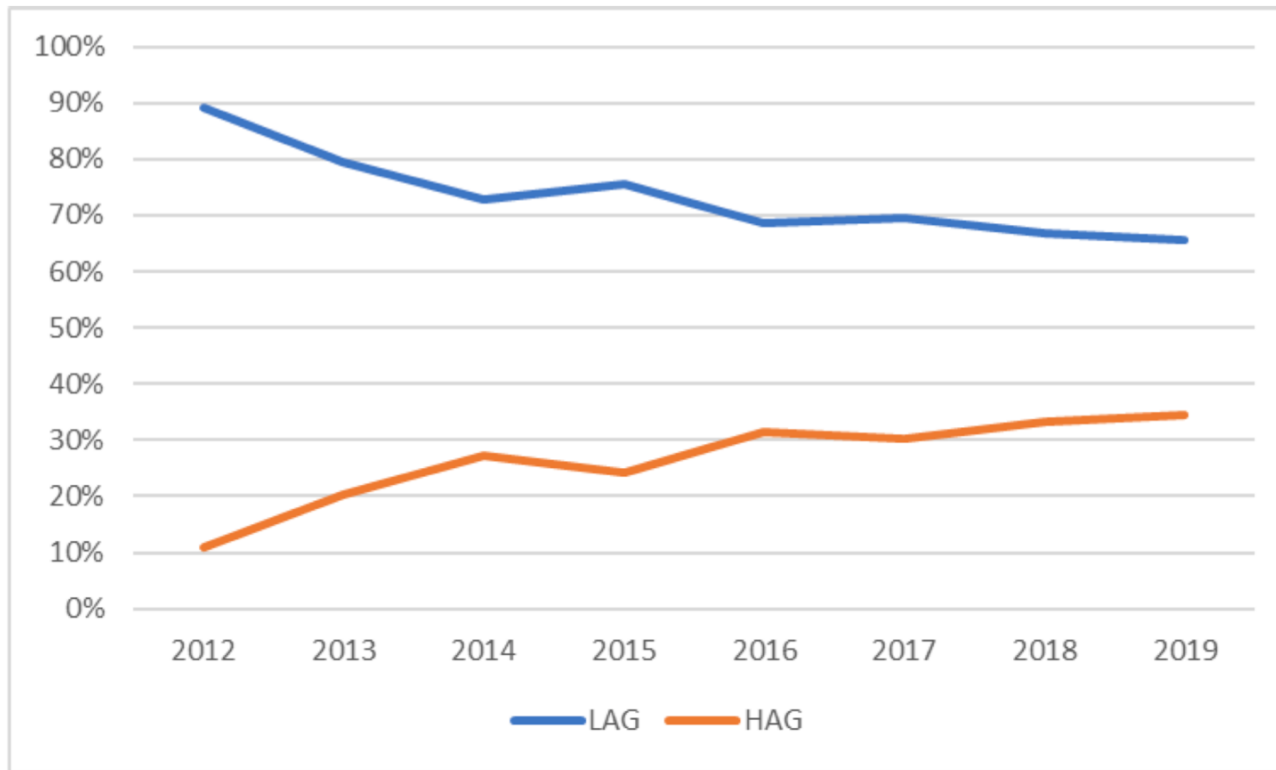

Supplement: S3 Fig — (PDF) [file pone.0308948.s003.pdf]
