# Supplementary material for: The impact of adhering to a quality indicator for sedation, analgesia, and delirium management on costs, revenues, and clinical outcomes in intensive care in Germany: A retrospective observational study
Source: PLoS One. 2024 Aug 15;19(8):e0308948. doi: 10.1371/journal.pone.0308948 (PMC11326618; doi:10.1371/journal.pone.0308948)
Supplement: S4 Fig — (PDF) [file pone.0308948.s004.pdf]

S4 Fig. Results for economic outcome following propensity score matching (n=4633)

a) DRG income

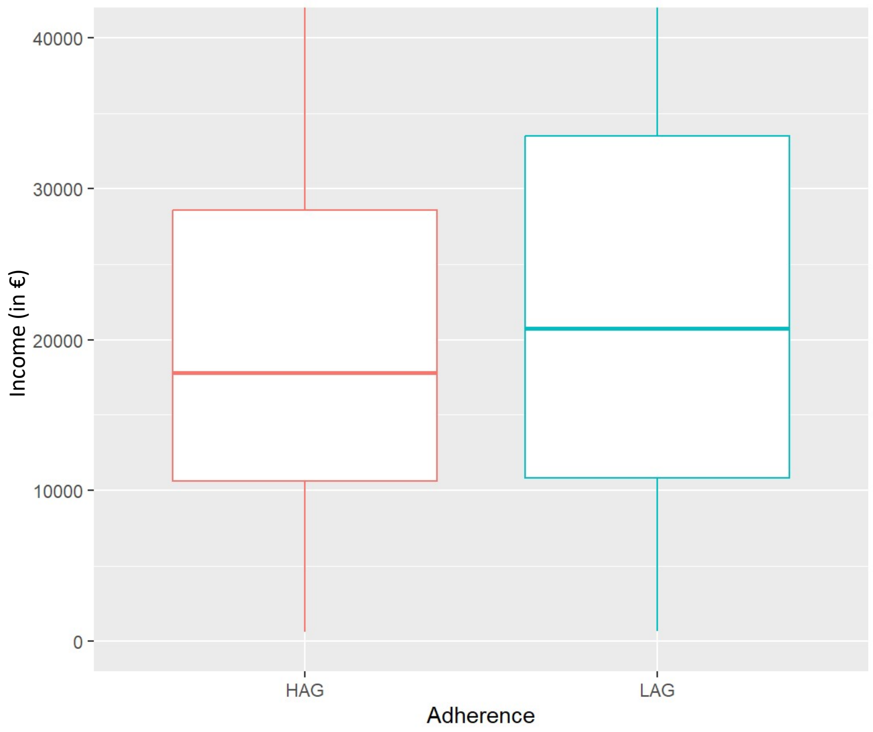

Q75	28575.0	33484.0
Median	17778.3	20750.9
Q25	10621.3	10810.4
Mean	23522.9	28363.5

b) Overall cost

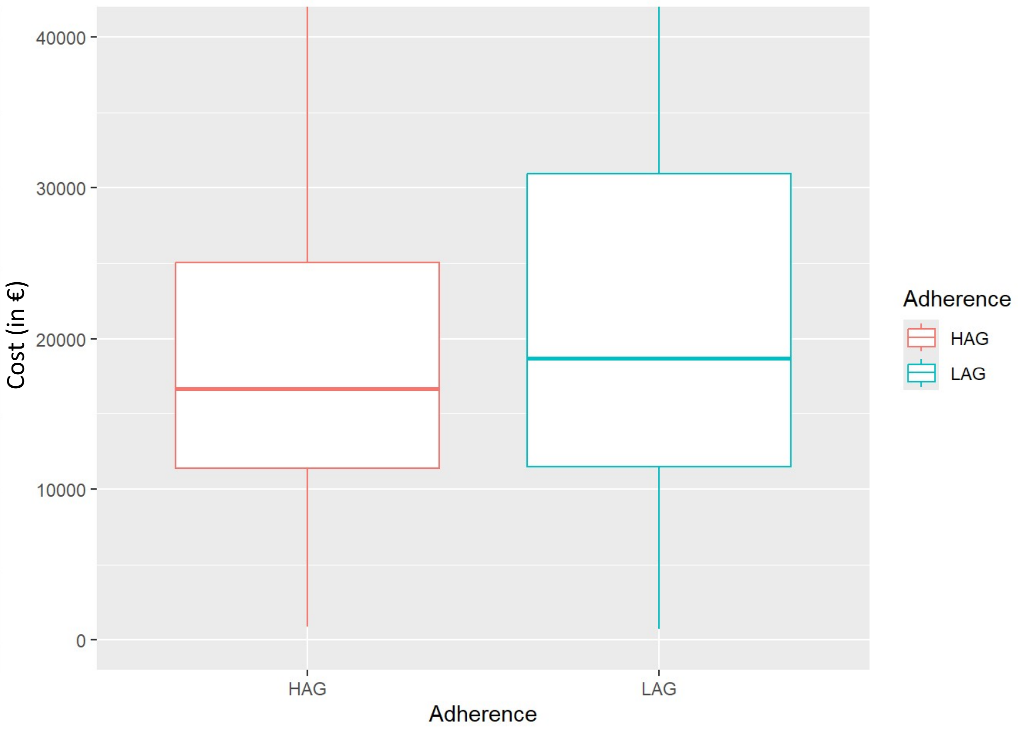

Q75	25076.1	30920.9
Median	16670.4	18696.0
Q25	11385.8	11493.8
Mean	22354.1	27281.7

Figure legend: Economic data of n=4633 patients following propensity score matching. Data shown as median with IQR. All numerical values in € (Euro). HAG = high adherence group, LAG = low adherence group
