# Supplementary material for: The impact of adhering to a quality indicator for sedation, analgesia, and delirium management on costs, revenues, and clinical outcomes in intensive care in Germany: A retrospective observational study
Source: PLoS One. 2024 Aug 15;19(8):e0308948. doi: 10.1371/journal.pone.0308948 (PMC11326618; doi:10.1371/journal.pone.0308948)
Supplement: S1 Table — (PDF) [file pone.0308948.s005.pdf]

S1 Table. Influence factors for high adherence (multiple logistic regression)

	HAG	
Predictors	*Odds Ratios*	*CI*
(Intercept)	0.28	0.22 – 0.35
Age	1.00	0.99 – 1.00
Male gender	1.11	1.03 – 1.19
SAPS-2 on admission	1.00	1.00 – 1.00
CCI (Age adjusted)	0.98	0.97 – 0.98
*Admission type**		
Emergency Surgery	0.54	0.49 – 0.59
Medical	0.43	0.40 – 0.47
*Main diagnosis category***		
Infection, sepsis	0.72	0.59 – 0.87
Malignant	0.87	0.78 – 0.97
Pulmonary	0.83	0.70 – 0.97
Other	0.68	0.62 – 0.75
Trauma	0.93	0.80 – 1.08
Cerebral	0.76	0.69 – 0.85
*Treatment year****		
2013	1.78	1.50 – 2.11
2014	2.46	2.07 – 2.94
2015	2.06	1.74 – 2.45
2016	3.21	2.73 – 3.78
2017	3.47	2.93 – 4.11
2018	3.67	3.12 – 4.33
2019	3.99	3.40 – 4.70
Observations	20220	
R^2 Tjur	0.062	

*reference: elective surgery; **reference: cardiac; ***reference: 2012
