# Supplementary material for: The impact of adhering to a quality indicator for sedation, analgesia, and delirium management on costs, revenues, and clinical outcomes in intensive care in Germany: A retrospective observational study
Source: PLoS One. 2024 Aug 15;19(8):e0308948. doi: 10.1371/journal.pone.0308948 (PMC11326618; doi:10.1371/journal.pone.0308948)
Supplement: S2 Table — (PDF) [file pone.0308948.s006.pdf]

S2 Table. Influence factors on overall cost (linear regression)

	Overall cost	
Predictors	*Estimate (€)*	*CI*
(Intercept)	26980.28	24449.17 – 29,511.38
High adherence group	-4849.28	-5841.58 – -3856.99
Age	-384.59	-414.55 – -354.62
Male gender	2530.16	1682.42 – 3377.91
SAPS-2 on admission	292.23	265.89 – 318.56
CCI (Age adjusted)	2437.11	2327.14 – 2547.07
Admission type*		
Emergency Surgery	6055.28	4815.16 – 7295.40
Medical	-996.61	-2027.97 – 34.74
Main diagnostic category**		
Infection, sepsis	1910.55	-317.36 – 4138.45
Malignant	-8658.01	-10087.76 – -7228.25
Pulmonary	7406.20	5464.58 – 9347.82
Other	208.20	-1039.97 – 1456.38
Trauma	-4283.46	-6237.62 – -2329.29
Cerebral	-7770.51	-9090.96 – -6450.05
Observations	20220	
R^2 / R^2 adj.	0.159 / 0.158	

*reference: elective surgery; **reference: cardiac
