# Supplementary material for: The impact of adhering to a quality indicator for sedation, analgesia, and delirium management on costs, revenues, and clinical outcomes in intensive care in Germany: A retrospective observational study
Source: PLoS One. 2024 Aug 15;19(8):e0308948. doi: 10.1371/journal.pone.0308948 (PMC11326618; doi:10.1371/journal.pone.0308948)
Supplement: S3 Table — (PDF) [file pone.0308948.s007.pdf]

S3 Table. Influence factors on length of hospital stay (linear regression)

	LOS hospital	
Predictors	*Estimate (days)*	*CI*
(Intercept)	17.68	16.21 – 19.14
High adherence group	-1.92	-2.49 – -1.35
Age	-0.15	-0.17 – -0.14
Male gender	0.68	0.19 – 1.17
SAPS-2 on admission	0.09	0.08 – 0.11
CCI (Age adjusted)	1.32	1.26 – 1.38
Admission type*		
Emergency Surgery	1.85	1.13 – 2.57
Medical	-0.43	-1.03 – 0.17
Main diagnostic category**		
Infection, sepsis	6.55	5.27 – 7.84
Malignant	1.65	0.82 – 2.47
Pulmonary	2.48	1.36 – 3.61
Other	4.98	4.26 – 5.70
Trauma	1.41	0.29 – 2.54
Cerebral	-2.61	-3.37 – -1.84
Observations	20220	
R^2	0.140	

*reference: Elective surgery; **reference: cardiac
