# Supplementary material for: The impact of adhering to a quality indicator for sedation, analgesia, and delirium management on costs, revenues, and clinical outcomes in intensive care in Germany: A retrospective observational study
Source: PLoS One. 2024 Aug 15;19(8):e0308948. doi: 10.1371/journal.pone.0308948 (PMC11326618; doi:10.1371/journal.pone.0308948)
Supplement: S4 Table — (PDF) [file pone.0308948.s008.pdf]

S4 Table. Cost items per case in € after propensity score matching (n=4633)

Cost item	**HAG**	**LAG**	p-value
Personnel	8404.98 [5624.86, 12376.96]	8617.53 [5289.97, 14660.54]	0.006
Infrastructure	4167.41 [2786.15, 6329.05]	4398.70 [2770.75, 7126.12]	<0.001
Material	3600.39 [2009.52, 6265.97]	4174.71 [2162.73, 9158.63]	<0.001
ICU	4512.20 [2210.75, 8681.63]	4688.18 [2152.60, 10897.25]	0.003

All values median and interquartile range
