# Supplementary material for: The impact of adhering to a quality indicator for sedation, analgesia, and delirium management on costs, revenues, and clinical outcomes in intensive care in Germany: A retrospective observational study
Source: PLoS One. 2024 Aug 15;19(8):e0308948. doi: 10.1371/journal.pone.0308948 (PMC11326618; doi:10.1371/journal.pone.0308948)
Supplement: S5 Table — (PDF) [file pone.0308948.s009.pdf]

S5 Table. Results for economic and clinical outcome following propensity score matching (n=4633)

a) Comparison of positive and negative revenue margins

	Positive or even margin	Negative margin
**HAG**	2413 (52.1)	2220 (47.9)
**LAG**	2432 (52.5)	2201 (47.5)

Pearson's Chi-squared test with Yates' continuity correction ($p=0.7081$)

b) Comparison of in-hospital mortality

	Died	Survived
**HAG**	404 (8.7)	4229 (91.3)
**LAG**	534 (11.5)	4099 (88.5)

Pearson's Chi-squared test with Yates' continuity correction ($p=0.009$)
