# Supplementary material for: The impact of adhering to a quality indicator for sedation, analgesia, and delirium management on costs, revenues, and clinical outcomes in intensive care in Germany: A retrospective observational study
Source: PLoS One. 2024 Aug 15;19(8):e0308948. doi: 10.1371/journal.pone.0308948 (PMC11326618; doi:10.1371/journal.pone.0308948)
Supplement: S6 Table — Algorithm used: Nearest neighbor. (PDF) [file pone.0308948.s010.pdf]

S6 Table. Overview over the propensity score matching categories and results. Algorithm used: Nearest neighbour.

Parameter	**Means Treated**	**Means Control**	**Std. Mean Diff.**	**Var. Ratio**	**eCDF Mean**	**eCDF Max**	**Std. Pair Dist.**
Distance	0.2979910	0.2973870	0.0058429	1.0209578	0.0004976	0.0123348	0.0060044
Age	64.1911194	64.0359728	0.0103098	0.8735489	0.0154962	0.0337004	1.1450945
Female gender	0.3898678	0.3914097	-0.0031613	NA	0.0015419	0.0015419	0.9253701
Male gender	0.6101322	0.6085903	0.0031613	NA	0.0015419	0.0015419	0.9253701
SAPS-2 on admission	37.0656388	36.8037445	0.0152292	1.0190569	0.0053822	0.0255507	1.1030705
CCI (Age adjusted)	5.4986784	5.5006608	-.0004568	1.0894320	0.0057685	0.0284141	0.9594906
*Admission type**							
Emergency Surgery	0.5083700	0.5044053	0.0079306	NA	0.0039648	0.0039648	0.3471852
Emergency Surgery	0.1808370	0.1808370	0.0000000	NA	0.0000000	0.0000000	0.2625551
Medical	0.3107930	0.3147577	-0.0085666	NA	0.0039648	0.0039648	0.5311264
*Treatment year****							
2013	0.1030837	0.1019824	0.0036220	NA	0.0011013	0.0011013	0.4643348
2014	0.1052863	0.1046256	0.0021530	NA	0.0006608	0.0006608	0.5590537
2015	0.1226872	0.1246696	-0.0060424	NA	0.0019824	0.0019824	0.5807422
2016	0.1735683	0.1762115	-0.0069789	NA	0.0026432	0.0026432	0.7211526
2017	0.1277533	0.1246696	0.0092378	NA	0.0030837	0.0030837	0.6479625
2018	0.1707048	0.1740088	-0.0087813	NA	0.0033040	0.0033040	0.7265047
2019	0.1969163	0.1938326	0.0077544	NA	0.0030837	0.0030837	0.7056549
*Main diagnosis category***							
Cardiac	0.3645374	0.3658590	-0.0027459	NA	0.0013216	0.0013216	0.7221628
Infection, sepsis	0.0286344	0.0266520	0.0118864	NA	0.0019824	0.0019824	0.3077265
Malignant	0.1770925	0.1773128	-0.0005770	NA	0.0002203	0.0002203	0.7195070
Pulmonary	0.0515419	0.0535242	-0.0089660	NA	0.0019824	0.0019824	0.4492950
Other	0.1823789	0.1788546	0.0091264	NA	0.0035242	0.0035242	0.7072982
Trauma	0.0599119	0.0629956	-0.0129937	NA	0.0030837	0.0030837	0.4789091
Cerebral	0.1359031	0.1348018	0.0032138	NA	0.0011013	0.0011013	0.6305467

eCDF = empirical cumulative distribution function
